# Integrating single-cell with transcriptome-proteome Mendelian randomization reveals colorectal cancer targets

**DOI:** 10.1007/s12672-025-02636-7

**Published:** 2025-05-17

**Authors:** Song Wang, Xin Yao, Shenshen Li, Shanshan Wang, Xuyu Huang, Jing Zhou, Xiao Li, Jieying Wen, Weixuan Lan, Yunsi Huang, Hao Li, Yunlong Sun, Xiaoqian Zhao, Qiaoling Chen, Xuedong Han, Ziming Zhu, Xinyue Zhang, Tao Zhang

**Affiliations:** 1 Guangxi University of Chinese Medicine, Nanning, Guangxi China; 2 Department of Gastroenterology, Ruikang Hospital of Guangxi Traditional Chinese Medical University, Nanning, Guangxi China; 3 Department of Oncology, Nanyang Hospital of Traditional Chinese Medicine, Nanyang, Henan China; 4 Department of Proctology, Chongqing Hospital of Traditional Chinese Medicine, Chongqing, Sichuan China

**Keywords:** Single-cell transcriptome, Mendelian randomization, Colorectal cancer, Gene

## Abstract

**Background:**

Colorectal carcinogenesis involves dynamic interactions between genetic susceptibility and cellular heterogeneity, yet current studies rarely disentangle causal genes from passive associations. While GWAS have mapped numerous risk loci, only a minority colocalize with eQTL/pQTL. A multi-omics framework combining single-cell transcriptomics, transcriptomics, proteomics, and MR is urgently needed to resolve cell-type-specific drivers of colorectal cancer pathogenesis.

**Methods:**

We integrated GWAS data, eQTL data, pQTL data, and single-cell RNA sequencing differential gene expression profiles from public databases. Subsequent batch Two-sample Mendelian randomization and further SMR analysis aimed to identify key genes in the pathogenesis of colorectal cancer.

**Results:**

Cluster analysis identified 4909 DEGs across various cell types. We discovered that 428 DEGs had a causal association with colorectal cancer through eQTL, of which 38 genes met the FDR statistical standards, and four of these genes (CTSF, PCSK7, LYZ, LMAN2L) also had causal associations through pQTL. SMR analysis confirmed the reliability of PCSK7 as a disease target.

**Conclusion:**

By integrating single-cell data, transcriptomic data, proteomic data and GWAS data for MR analysis, we identified CTSF, PCSK7, LYZ, LMAN2L as potential targets for colorectal cancer.

**Supplementary Information:**

The online version contains supplementary material available at 10.1007/s12672-025-02636-7.

## Introduction

Colorectal cancer (CRC) has emerged as one of the most prevalent malignant tumors in the digestive system, with its incidence showing a persistent upward trend globally. Although traditional epidemiological studies have identified major modifiable risk factors, including Western dietary patterns, sedentary lifestyles, and excessive alcohol consumption/smoking, there remains a significant gap in understanding how genetic susceptibility interacts with microenvironmental perturbations to drive colorectal carcinogenesis [1, 2]. This knowledge deficiency has somewhat constrained the development of precision prevention strategies and targeted therapies.

Recent advances in multi-omics technologies are beginning to redefine our understanding of CRC pathogenesis. While genome-wide association studies (GWAS) have identified numerous genetic susceptibility loci, these variants collectively explain only a relatively small proportion of heritable risk, suggesting the existence of complex mechanisms underlying gene-environment interactions. Concurrently, emerging single-cell transcriptomic analyses have mapped gene expression profiles at single-cell resolution within the tumor microenvironment, partially revealing dynamic cellular crosstalk among malignant epithelial cells, immune infiltrates, and stromal components [3]. However, existing research approaches remain relatively isolated methodologically: GWAS identifies risk loci but lacks cellular resolution, whereas single-cell analyses map cellular ecosystems but struggle to establish causal relationships with genetic drivers. Thus, integrating causal inference frameworks with high-resolution single-cell data presents a transformative opportunity.

Expression quantitative trait loci (eQTL) and proteome QTL (pQTL) analyses bridge the genotype–phenotype gap at transcriptional and translational levels [4, 5]. Mendelian randomization (MR), leveraging genetic variants as instrumental variables, overcomes confounding biases inherent in observational studies and enables causal estimation of exposure-disease relationships [6]. Furthermore, summary data-based Mendelian randomization (SMR) analysis enhances the precision of causal inference [7]. Through systematic integration of GWAS findings with single-cell transcriptomic atlases, we have uncovered novel therapeutic targets while establishing an analytical framework that translates genetic associations into actionable biological insights. These discoveries not only advance our understanding of colorectal carcinogenesis but also provide a roadmap for developing personalized prevention strategies and advancing precision oncology.

## Methods and materials

### Colorectal cancer GWAS data source

This study obtained clinical and genetic data related to colorectal cancer from the Finnish nationwide cohort study database [8] (https://www.finngen.fi/en). As a large-scale, long-term follow-up population cohort study platform, it provided us with high-quality, representative research samples. Through strict data screening and quality control processes, we extracted a detailed dataset including genetic variant information, encompassing 8,801 cases and 45,118 controls.

### Single-cell transcriptomics data source

We downloaded colorectal cancer-related single-cell transcriptomic sequencing datasets from the Gene Expression Omnibus (GEO) database (GSE200997), which includes samples from 16 tumor tissues and 7 normal tissues. Each sample was processed using DAPI-FACS buffer after enzymatic digestion with Miltenyi, and single-cell capture and sequencing were performed using the 10X Genomics Single Cell 5′ platform. Library construction and sequencing were completed on an Illumina NextSeq 550, and the data were analyzed using Cellranger-v3.1.0. This scRNA-seq delineated a detailed map of CRC tumors and their microenvironment, revealing a complex landscape of cell diversity and heterogeneity across different patient groups. To strengthen the validation of our findings, we incorporated an additional single-cell RNA sequencing dataset (GSE166555) for external verification. This dataset comprises tumor tissues and adjacent normal tissues collected from 12 surgical resection patients, processed through mechanical/enzymatic dissociation into single-cell suspensions. The scRNA-seq libraries were generated using the Chromium Controller (10 × Genomics) with 3′ v2 chemistry, following standardized droplet-based protocols. This well-characterized dataset provides an independent validation cohort with balanced tumor-normal sampling, enabling robust cross-platform confirmation of our analytical outcomes.

### Genetic variant data acquisition

The eQTLGen Consortium, a large-scale international collaboration, investigates the role of eQTL in complex traits and diseases. The consortium has compiled extensive gene expression and genotype data from multiple research centers, covering various populations and tissue types. By utilizing eQTLGen data, researchers can conduct systematic MR analysis to infer the causal role of gene expression in diseases. The original data for pQTL came from a large-scale GWAS dataset published on the decode website [9]. The team used the fourth version of the SomaScan multiplex aptamer detection technology to analyze genetic and phenotypic data from 35,559 Icelanders. The study analyzed associations among 4907 plasma proteins, 27.2 million genomic sequence variations, and 373 diseases/traits. Whole-genome sequencing and genotyping revealed 18,084 principal pQTL associations: 1881 cis-effects (proximal to gene regions) and 16,203 trans-effects (distal regulatory effects). These regulatory loci covered 94% of the studied proteins, revealing the complexity of gene expression regulation. Additionally, the statistical processing of the study ensured only a 1.3% false discovery rate, providing high reliability scientific evidence for proteomics research.

### Single-cell transcriptomics data analysis

We performed data import and preprocessing using the OmicsTools bioinformatics software and conducted quality control to remove low-quality data. Cells with a mitochondrial RNA (mtRNA) proportion greater than 10% or with fewer than 200 or more than 5000 genes were filtered out. Subsequently, we integrated data from different samples for normalization and principal component analysis (PCA). Using RNA expression data, we performed cell clustering and dimensionality reduction visualization, compared cell-specific gene expression differences under various conditions, and followed by functional enrichment analyses (GO and KEGG) to characterize molecular pathways and biological processes associated with differentially expressed genes (DEGs).

### Two-sample Mendelian randomization

To enhance the accuracy of causal inference, we further conducted two-sample Mendelian Randomization analysis. This approach employs genetic instruments to infer causal associations between transcriptomic and proteomic biomarkers and colorectal cancer. We identified cis-acting eQTL/pQTL-associated Single-nucleotide polymorphisms (SNPs) meeting genome-wide significance threshold (p < 5 × 10^ −8^) through GWAS; applied instrument selection criteria (r^2^ < 0.1, clumping distance > 10,000 kb) to remove correlated variants [10]. These SNPs, serving as significant instrumental variables, were used for subsequent analyses. Using the Two-Sample MR approach, we applied statistical methods (e.g., Inverse Variance Weighting [IVW] [11] and MR-Egger regression [12]) to estimate causal effects of significant SNPs, while correcting for pleiotropy and confounding. The F-value was utilized to assess the strength of the genetic instruments to ensure suitability for causal inference [13]. Only instruments with an F-value greater than 10 were considered strong and capable of robustly representing exposure factors, thus reducing biases and inaccuracies caused by weak instruments and enhancing the credibility of the analysis results. We further used the False Discovery Rate (FDR) test to ensure the robust causal association of selected SNPs with colorectal cancer. Mendelian randomization relies on three core principles [14]: (1) Association: Instrumental variants (IVs) must demonstrate robust correlation with target exposures. (2) Independence: IVs should remain unconfounded by measured or latent variables (e.g., environmental/socioeconomic factors). (3) Exclusivity: IVs exert effects exclusively through exposure pathways without pleiotropic mechanisms. All aforementioned MR analytical methods were implemented in January 2025 using the TwoSampleMR package (version 0.6.6) within R statistical software (version 4.4.1).

### Summary-data-based Mendelian randomization analysis

**Fig. 1:**
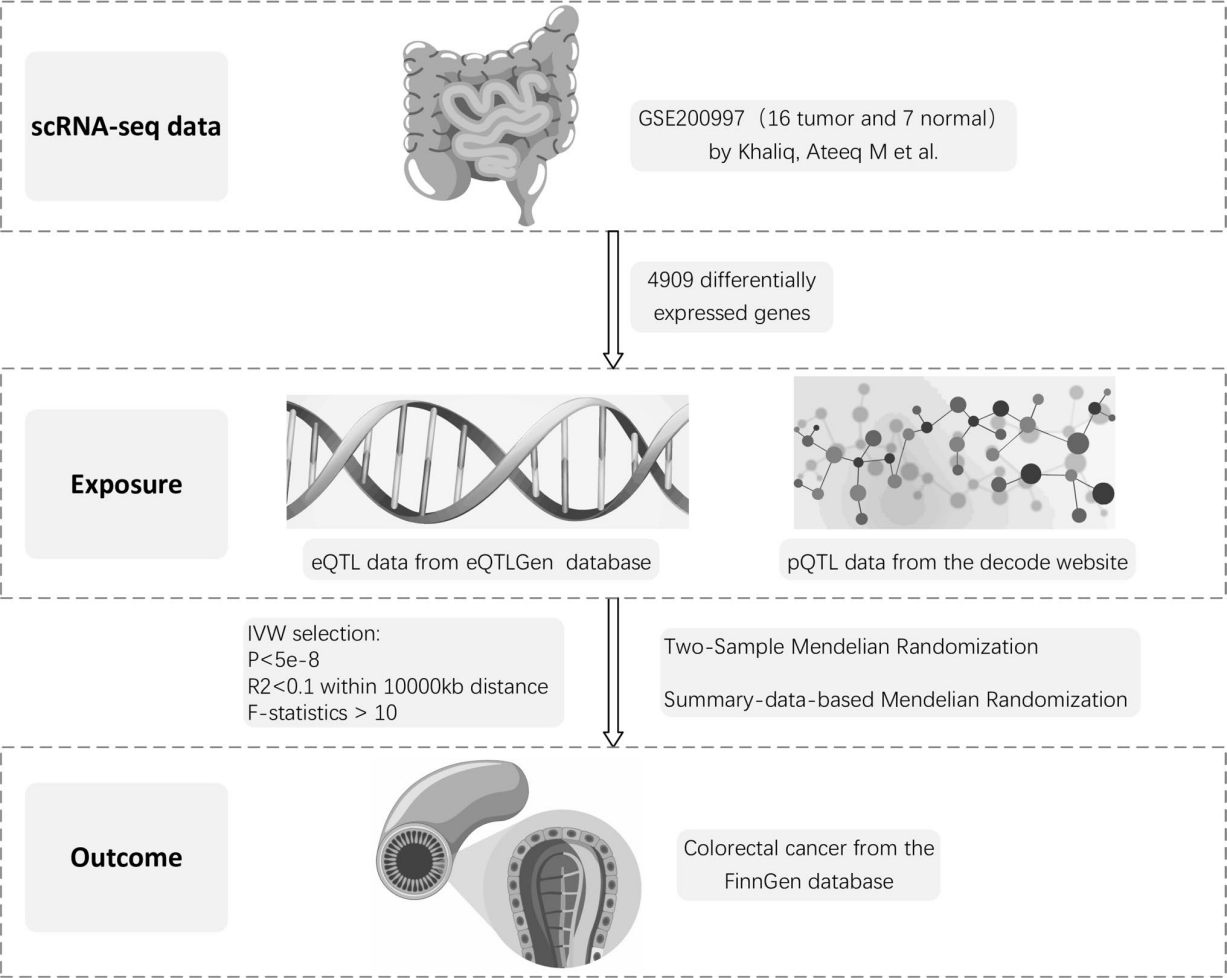
Single-cell analysis and Mendelian randomization analysis design flowchart. scRNA-se: single-cell RNA sequencing; eQTL: expression quantitative trait loci; pQTL: protein quantitative trait loci

SMR applies genetic variants as instrumental tools to establish causal associations between gene expression patterns and disease outcomes. The HEIDI heterogeneity test evaluates SNP pleiotropy affecting both target genes and disease phenotypes, differentiating true causation from linkage disequilibrium effects to validate SMR findings [15]. Significant heterogeneity suggests the potential influence of other pathways or pleiotropic effects. The flowchart of this study is illustrated in Fig. 1.

### Survival analysis

We integrated COAD (Colon Adenocarcinoma) and READ (Rectum Adenocarcinoma) datasets from the The Cancer Genome Atlas (TCGA) database, consolidating them into a unified colorectal cancer cohort for survival analysis. To evaluate the prognostic potential of candidate genes in colorectal cancer, a risk score model was constructed using multivariate Cox proportional hazards regression analysis. The risk score for each patient was calculated as follows: Risk Score = Σ(β_i_ × Gene_i_), where β_i_ represents the regression coefficient derived from Cox analysis and Gene_i_ corresponds to the normalized expression level of each gene. Patients were stratified into high-risk and low-risk groups based on the median risk score. Univariate and multivariate Cox regression analyses were performed to assess the independence of the risk score as a prognostic factor, with covariates including age, tumor stage, and other clinicopathological parameters. The predictive accuracy of the risk score for 1-, 3-, and 5-year survival was quantified using time-dependent receiver operating characteristic (ROC) curves. Kaplan–Meier survival curves were generated to compare survival outcomes between risk groups, and log-rank tests were employed to validate statistical significance (p < 0.05). All survival analyses were performed in March 2025 using the survival package (version 3.7-0) within R software.

## Results

### Changes in colorectal cancer cell subpopulations in tumor and non-tumor environments

**Fig. 2:**
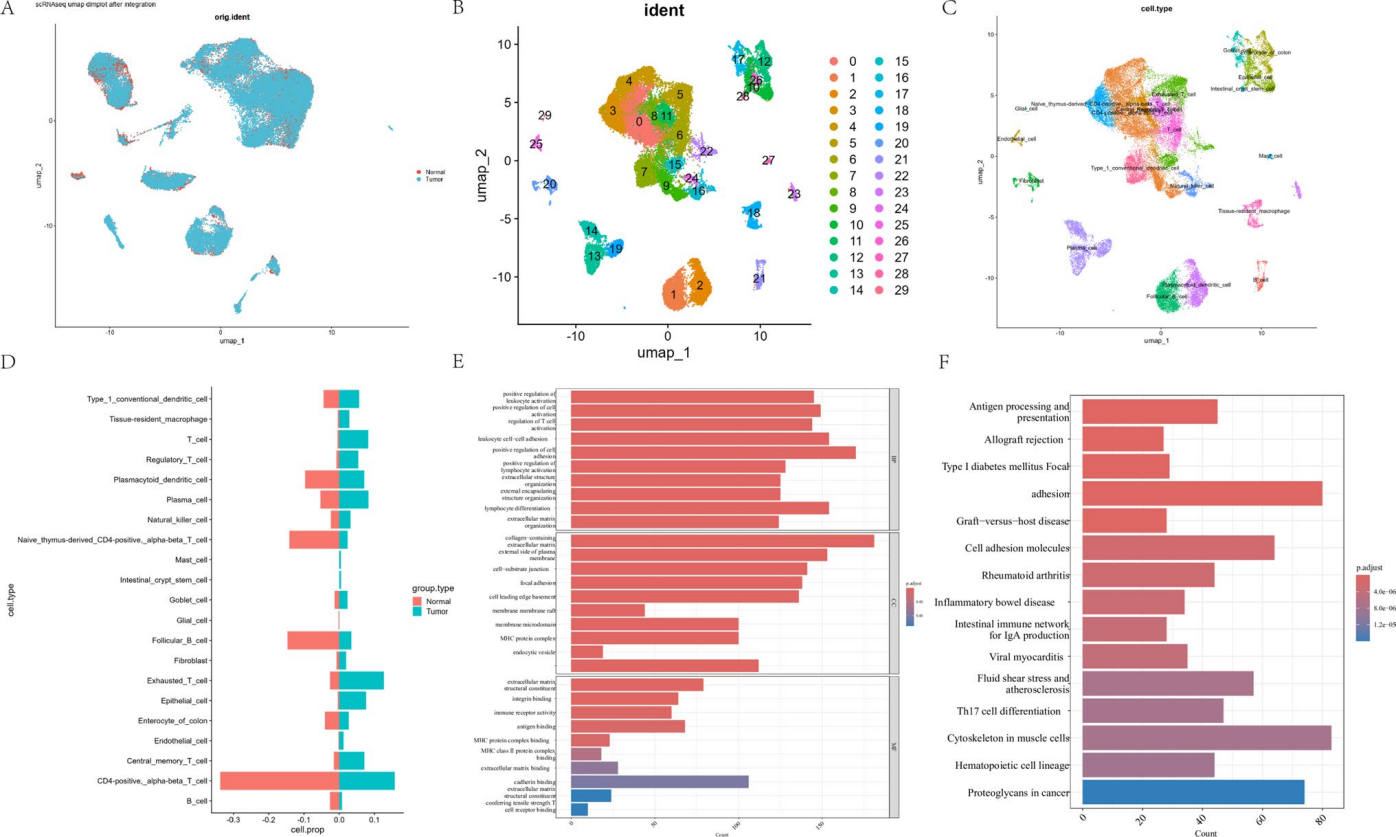
Processing of single-cell sequencing data and analysis of differentially expressed genes (from GSE200997). **A** Removal of batch effects; **B** Clustering analysis; **C** Cell type annotation; **D** Differential expression of cell subtypes; **E**, **F** GO analysis and KEGG analysis of differentially expressed genes

In this study, we analyzed transcriptomic sequencing data from colorectal cancer tissues of 16 donors and non-tumor tissues of 7 donors (GSE200997). After filtering low-quality cells and genes, we integrated datasets from different samples and corrected batch effects (Fig. 2A). This yielded 18,273 normal tissue cells and 31,856 cancer tissue cells. We performed cell clustering using UMAP and visualized the results, revealing 29 cluster categories (Fig. 2B), 10 cell types (Fig. 2C), and the proportion differences of 21 different cell subtypes in each sample or condition (Fig. 2D). Subsequently, we used |logFC|≥ 1 and p < 0.01 as screening criteria to integrate DEGs from 21 cell subtypes. After removing duplicates, we obtained 4,909 DEGs (see Supplementary Table S1). Functional annotation showed that DEGs were primarily involved in immune cell activation, cell adhesion, extracellular matrix organization, and immune-related disease pathways (Fig. 2E, F).

### Causal association analysis of eQTL in colorectal cancer

**Fig. 3:**
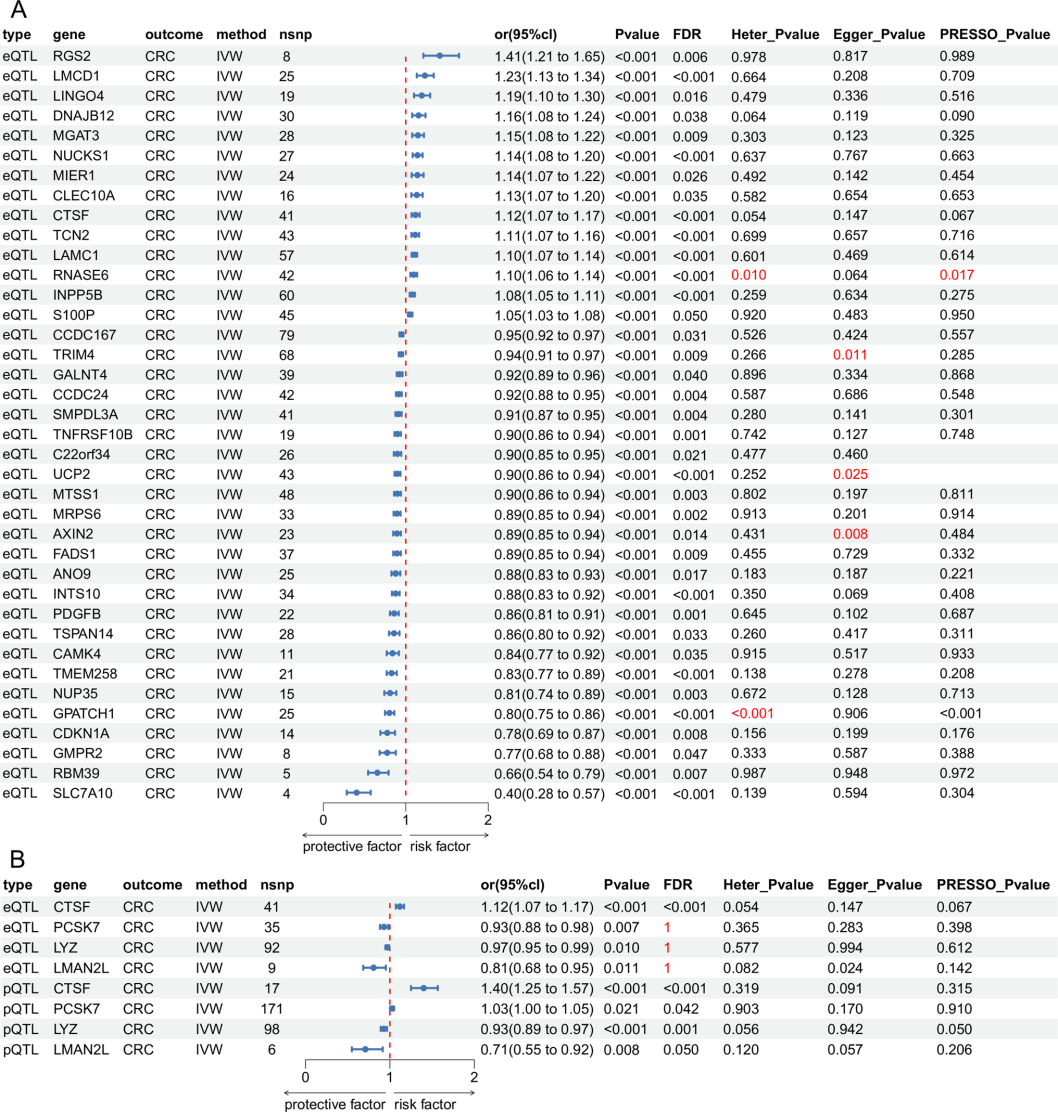
Forest plot visualization of Mendelian randomization analysis. **A** 38 eQTL genes meet the FDR statistical threshold; **B** 4 pQTL genes meet the FDR statistical threshold, and their results in the eQTL analysis are also shown

We integrated the preprocessed eQTL with 4909 DEGs and performed MR analysis to explore potential associations with CRC. We successfully identified 428 genes showing significant genetic associations with colorectal cancer. Supplementary Table S2 presents all eQTL analysis outcomes demonstrating statistically significant associations. Only 38 eQTL genes met the statistical criterion of a FDR less than 0.05, among which 24 genes were negatively correlated with CRC risk, and 14 genes increased CRC risk. Sensitivity analysis via the Q-test suggested potential bias in the GPATCH1 and RNASE6 gene results. UCP2, TRIM4, and AXIN2 might influence the outcome variable through pathways other than the studied exposure factors. The visualization forest plot of the 38 positive results after FDR screening is shown in Fig. 3A.

### Causal association analysis of pQTL in colorectal cancer

To further reveal the causal associations between the proteins encoded by these genes and CRC, we performed MR analysis of pQTL for the 428 positive result genes. We finally obtained 13 positive results, of which only 4 genes met the FDR statistical criterion (see Supplementary Table S3). Among them, the eQTL and pQTL directions were consistent for CTSF, LYZ, and LMAN2L, while only PCSK7 had an inconsistent direction. Except for PCSK7, LYZ, and LMAN2L, which failed the FDR test, the remaining results had an FDR less than 0.05. All results showed no heterogeneity or pleiotropy. As systematically illustrated in Fig. 3B, ‘Type’ and ‘Gene’ delineate the study subject categories and genetic loci respectively, while ‘Outcome’ and ‘Method’ specify the clinical endpoints and analytical approaches. ‘nsnp’ indicates the number of incorporated genetic instruments. ‘or(95%cI)’ quantifies exposure-outcome association magnitude with corresponding confidence intervals, complemented by ‘pvalue’ denoting statistical significance. Multiple testing correction is implemented through ‘FDR’. Methodological robustness is systematically verified by: (1) ‘Heter_Pvalue (Cochran’s Q test)’ evaluating between-SNP heterogeneity; (2) ‘Egger_Pvalue (MR-Egger intercept test)’ assessing directional pleiotropy; and (3) ‘PRESSO_Pvalue (MR-PRESSO)’ detecting and correcting for outlier variants. This multi-layered sensitivity framework ensures result reliability through complementary pleiotropy-resistant approaches.

### SMR validation analysis

**Fig. 4:**
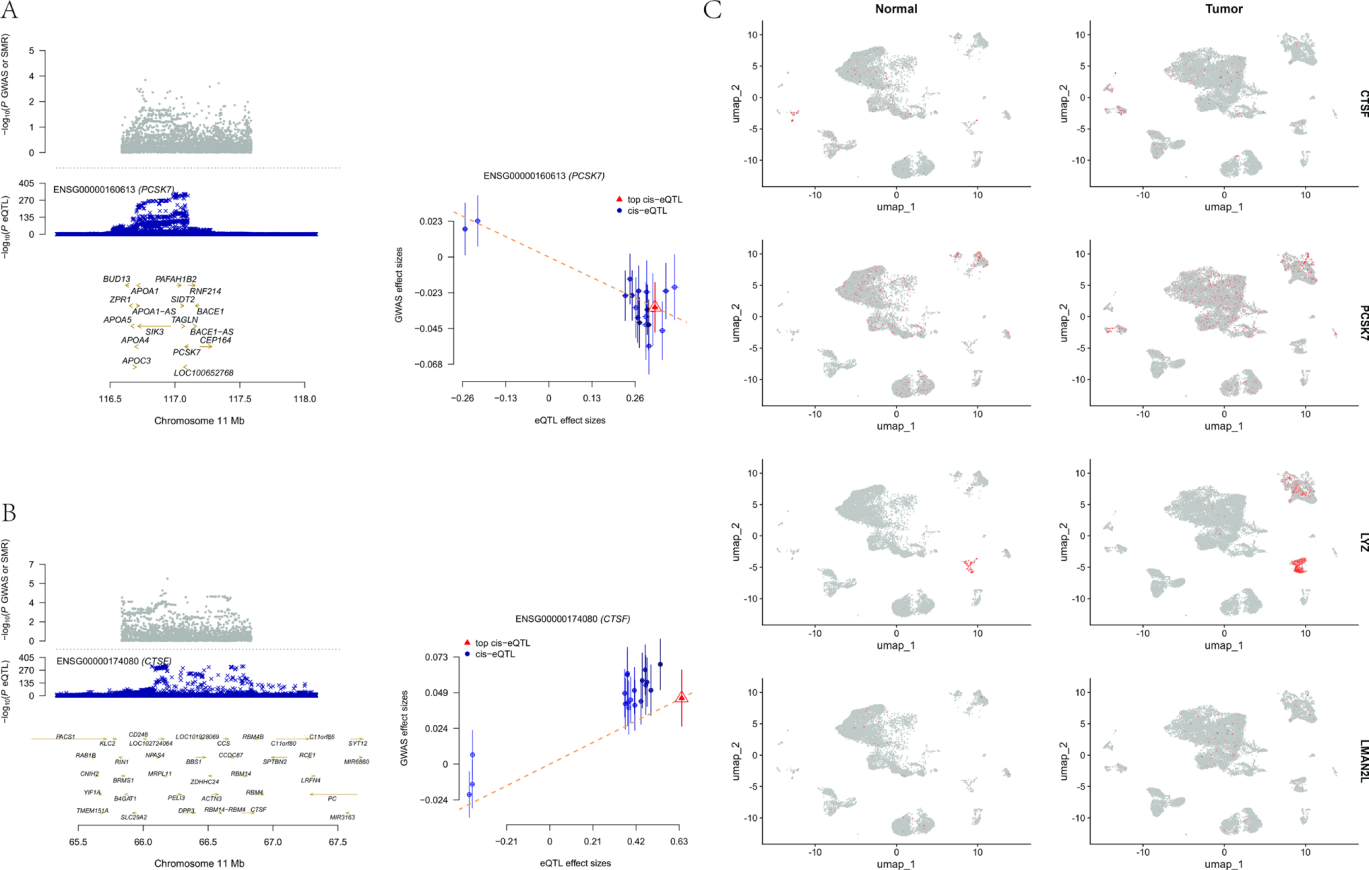
SMR validation analysis and differential expression analysis of hub genes at the single-cell level. **A**, **B** Forest plot visualization of SMR analysis; **C** Differential expression of 4 hub genes across different tissues (from GSE200997)

We performed further SMR validation analysis on the 4 genes that simultaneously met the eQTL and pQTL positive criteria and passed the FDR statistic (< 0.05) (see Supplementary Table S4). The results showed that the gene expression of PCSK7 (OR = 0.90, 95%CI 0.82–0.99, pSMR = 0.0426, pHEIDI = 0.429) was a protective factor for CRC, while the gene expression of CTSF (OR = 1.07, 95%CI 1.01–1.14, pSMR = 0.0184, pHEIDI = 0.005) was a risk factor for CRC. The HEIDI test suggested that CTSF-associated SNPs may affect outcomes via alternative pathways. The visualization forest plot of the SMR analysis is shown in Fig. 4A, B.

### Single-cell differential expression

Our cross-cohort analysis revealed conserved and divergent expression patterns for tumor-associated genes. Consistently, CTSF exhibited significant downregulation in fibroblasts (avg_log2 FC: GSE200997 = − 1.06; GSE166555 = − 0.67), plasma cells (− 0.93 vs. − 0.92), and tissue-resident macrophages (− 3.04 vs. − 1.71) across both cohorts (p < 0.01). PCSK7 was upregulated in fibroblast lineages (2.59 vs. 0.14, p < 0.01), and LYZ showed marked upregulation in epithelial cells in both datasets (1.10 vs. 2.38, p < 0.01). Notable inconsistencies highlighted microenvironmental heterogeneity. CTSF suppression in central memory T cells was specific to GSE200997 (− 1.69, p < 0.01), with no significant alteration in GSE166555 (0.10, p = 0.64). LYZ displayed opposing endothelial cell regulation (− 3.64 vs. 1.47, p < 0.05), while LMAN2L downregulation in endothelial cells (− 4.41, p < 0.01) was exclusive to GSE200997. Also, LMAN2L upregulation in exhausted T cells (1.40, p < 0.05) observed in GSE200997 failed to replicate in the validation cohort (0.73, p = 0.55). Divergent LYZ and LMAN2L endothelial trends, coupled with cohort-specific CTSF dynamics in T cell subsets, underscore spatial or molecular heterogeneity in tumor microenvironments. These findings emphasize context-dependent gene dysregulation in malignancy and advocate multi-cohort validation for stromal-immune biomarkers. Tissue differential gene expression is shown in Fig. 4C (from GSE200997), Supplementary Figure S1 (from GSE166555) and Supplementary Table S5.

### Survival analysis

**Fig. 5:**
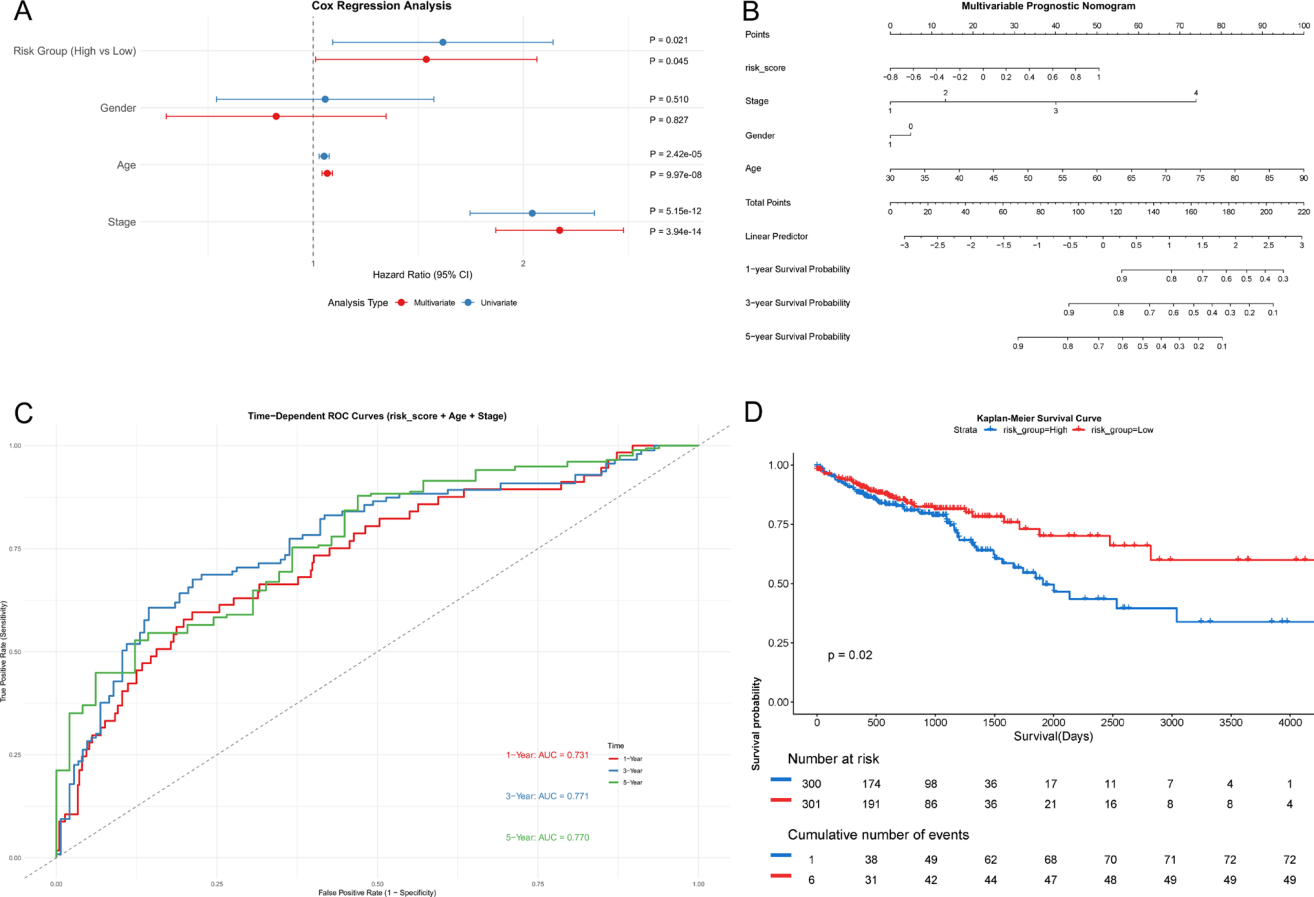
Survival analysis of patients in different risk groups. **A** Forest plot of univariate and multivariate Cox regression analyses. **B** Multivariable Prognostic Nomogram. **C** Time-dependent ROC curves. **D** Kaplan–Meier curves of different risk groups

By leveraging a four-gene expression signature, colorectal cancer patients were stratified into high- and low-risk subgroups. Multivariate Cox regression analysis identified the risk score as an independent prognostic factor (p < 0.05), with consistent significance in univariate analysis (p < 0.05) (Fig. 5A). The developed nomogram (Fig. 5B) integrates four prognostic parameters on parallel axes: risk score (− 0.8 to 1), tumor stage (I-IV), gender (0/1), and age (30–90 years), enabling conversion of total points (0–220) to 1-, 3-, and 5-year survival probabilities (0.1–0.9) through linear predictor values (− 3 to 3). Time-dependent ROC curves demonstrated robust predictive accuracy for 1-, 3-, and 5-year survival (AUC = 0.731, 0.771, and 0.770, respectively) (Fig. 5C). Kaplan–Meier survival analysis further corroborated that high-risk patients exhibited significantly poorer overall survival compared to the low-risk cohort (p < 0.05) (Fig. 5D). These findings underscore the clinical utility of this gene-based risk model in prognostication and personalized therapeutic strategies for CRC.

## Discussion

By integrating single-cell clustering data, eQTL, pQTL data, and GWAS data for colorectal cancer, we conducted two-sample Mendelian randomization and SMR analyses, identifying statistically significant associations between changes in the expression levels of four genes (CTSF, PCSK7, LYZ, LMAN2L) and susceptibility to CRC.

CTSF (Cathepsin F), a lysosomal protease family member, participates in protein breakdown and autophagy, contributing to immune regulation [16], programmed cell death, and tumor microenvironment modulation. Studies show that CTSF expression is significantly reduced at both mRNA and protein levels in clear cell renal cell carcinoma (ccRCC), which correlates with poor clinical outcomes. Experimental studies demonstrate that elevating CTSF expression effectively suppresses growth and metastatic potential in 786-O and 769-P cell lines [17]. Similarly, gastric cancer exhibits notable reduction of CTSF levels in both cellular models and clinical tissue samples. After knocking down CTSF expression using CTSF-Lenti-shRNA, there was a notable increase in GC cell growth and a simultaneous reduction in apoptosis levels, further confirming CTSF’s tumor-suppressive function [18]. In cervical cancer, studies have found that CTSF gene expression is markedly upregulated, and its reliability has been validated using endpoint RT-PCR technology [19]. Furthermore, in brain metastasis tissues from non-small cell lung cancer (NSCLC), CTSF expression is significantly higher compared to non-brain metastasis NSCLC and other primary brain tumors, with high consistency in CTSF expression between primary lung tumors and corresponding brain metastasis lesions, indicating that serum CTSF could serve as a specific biomarker for NSCLC brain metastasis [20]. Additionally, the anti-apoptotic effect of adipose-derived mesenchymal stem cells (ADSCs) is closely related to the downregulation of CTSF expression. By downregulating key pro-apoptotic mediators (e.g., Bid, BAX, caspase-9) while upregulating essential anti-apoptotic regulators (e.g., Bcl-2/Bcl-XL), ADSCs demonstrate dual anti-apoptotic and pro-regenerative capacities, effectively mitigating radiation-induced skin inflammation across both acute and chronic forms [21]. Our research also indicates that CTSF is a risk gene for colorectal cancer. Overall, CTSF has demonstrated potential as a disease biomarker and therapeutic target, but its function may vary across different types of tumors, and its mechanisms of action require further elucidation.

PCSK7 (Proprotein Convertase Subtilisin/Kexin Type 7), an enzyme within the proprotein convertase group, plays a role in the proteolytic processing and activation of precursor proteins. In lipid metabolism, individuals carrying the PCSK7 functional gain SNP (rs236918) showed elevated plasma triglycerides (TG), total cholesterol (TC), apolipoprotein B (apoB), and increased liver cirrhosis risk [22]. Research demonstrates PCSK7 facilitates apoB100 secretion through endoplasmic reticulum interaction, while its deficiency triggers proteasomal degradation of apoB100. This cascade activates the unfolded protein response (UPR), stimulates autophagic activity and fatty acid β-oxidation, culminating in diminished hepatic lipid deposition. In a mouse model, silencing Pcsk7 expression in hepatocytes through hepatocyte-specific N-acetylgalactosamine (GalNac) modified antisense oligonucleotides (ASOs) significantly improved the non-alcoholic fatty liver disease(NAFLD) phenotype. This treatment reduced plasma apoB levels by about 50%, while also lowering liver lipid content, inflammation, hepatocellular ballooning, and fibrosis [23]. In iron metabolism, in vitro experiments have shown that reducing PCSK7 expression using siRNA leads to a significant decrease in HAMP (encoding hepcidin) mRNA levels in liver cancer cells, confirming the impact of PCSK7 on the expression of the key iron metabolism regulator hepcidin, and suggesting that PCSK7 gene variations could be one of the important genetic factors leading to primary iron overload [24]. Emerging evidence indicates PCSK7 expression positively associates with T cell activation markers, potentially through modulating FoxP3 pre-mRNA splicing. Despite this regulatory linkage, spatial dissociation persists in atherosclerotic lesions, likely attributable to the limited Treg population within these pathological microenvironments [25]. Additionally, in the context of tumors, PCSK7 and Furin by cleaving CASC4 (a protein that maintains cell skeletal integrity) generate oncogenic N-terminal fragments that disrupt the cell skeleton, thereby affecting the malignant phenotypes such as migration and invasion in breast cancer cells [26]. Another study showed that in invasive colorectal cancer tissues, PCs activity is elevated, inducing high expression of molecules such as PD-1, leading to suppression of CD8 + T lymphocyte (CTL) function, preventing effective tumor cell clearance. However, using PCs inhibitors can reduce PD-1 expression, restoring and enhancing T cell anti-tumor immune response [27]. Our study results show directional inconsistency, with PCSK7’s eQTL and SMR analysis indicating it as a protective factor for CRC, yet the pQTL genes regulating it act as risk factors, suggesting that the complex biological functions of this protein may give it a dual role in CRC development. Future research needs to further explore the specific mechanisms of PCSK7 in colorectal cancer and how to prevent or treat CRC by modulating PCSK7 activity [28].

The LYZ gene encodes the lysozyme protein, an enzyme with antimicrobial activity that hydrolyzes peptidoglycan components in bacterial cell walls, thereby disrupting the cell wall and causing bacterial lysis and death. Studies have shown that *Fusobacterium nucleatum* (Fn) uses its virulence factor Fn1792 to resist intestinal lysozyme, promoting mucosal colonization and evading phagocytic lysosomal degradation. Phagocytes infected with Fn, after forming a special CX3 CR1 + PD-L1 + subpopulation, are continuously recruited to the tumor site. They reshape the tumor microenvironment by releasing enzymes like matrix metalloproteinase 9 (MMP-9) and signaling molecules such as vascular endothelial growth factor (VEGF), thereby accelerating tumor progression and metastasis. In summary, lysozyme inhibition is the initial step in this process, while CX3 CR1 and PD-L1 mediate the key steps in the transformation of phagocytes to a pro-tumor phenotype [29]. In an animal experiment with Axin1 gene knockout mice, it was found that the absence of the Wnt/β-catenin signaling pathway regulator TCF-1 leads to decreased lysozyme expression, accompanied by changes in goblet cell morphology and number, and increased secretion of MUC2 mucin. These changes promoted the enrichment of Akkermansia muciniphila, and despite the reduction in lysozyme, these mice showed greater resistance to dextran sulfate sodium (DSS)-induced colitis. These findings highlight the dual role of lysozyme in gut homeostasis. On one hand, as an antimicrobial peptide, it participates in innate immune defense, maintaining gut barrier integrity by inhibiting the growth of potential pathogens in the early stages of disease. On the other hand, through its regulatory effect on the gut microbiome, it shows important therapeutic potential for modulating gut inflammatory responses and preventing post-traumatic complications [30, 31]. Dietary restriction (DR) two weeks before chemotherapy can significantly prevent lysozyme loss, increase Lactobacillus content, thereby significantly inhibiting gut opportunistic pathogens and their translocation, and significantly improving the survival of elderly mice receiving 5-FU treatment [32]. Furthermore, exogenous lysozyme supplementation can not only alleviate the severity of acute pancreatitis (AP) and its intestinal complications by regulating the gut-pancreas axis [33], but also indirectly protect lung health through the gut-lung axis, reducing lung injury caused by oxygen excess [34]. Our research also indicates that LYZ is a protective factor for CRC. In the future, strategies such as dietary intervention and probiotic supplementation [35] can be further explored to increase LYZ gene expression and activity, and to evaluate its application prospects in CRC prevention and treatment.

The LMAN2L gene encodes a non-cycling resident protein in the endoplasmic reticulum (ER) that can specifically bind to mannose as a cargo receptor for glycoproteins in the ER, thereby participating in protein transport. It facilitates transport of correctly folded proteins to the Golgi apparatus and directs misfolded glycoproteins to the ubiquitin–proteasome pathway. This function may be related to the transport of neuroreceptors and ER stress, thus affecting the homeostasis and function of the nervous system [36, 37]. Evidence suggests that variations in this gene are associated with autosomal recessive mental retardation type 52 (MRT52), and these variations may lead to loss of protein function or changes in its sugar-binding affinity, thereby affecting its normal physiological functions. Patients can present with symptoms such as delayed brain development, severe intellectual disability, speech disorders, epilepsy, bipolar disorder, and schizophrenia [38–42]. Studies have shown that the protein encoded by LMAN2L can promote the phagocytosis of pathogens by macrophages and clear apoptotic cells. Downregulation of LMAN2L gene expression plays an important role in the pathogenesis of COPD with emphysema by weakening airway infection defense and apoptotic cell clearance mechanisms [43]. Similarly, human cytomegalovirus employs its pUS2 protein to dysregulate host ER-associated degradation processes, recruits the cellular E3 ubiquitin ligase TRC8 to ubiquitinate LMAN2L, affecting the expression of cell surface proteins dependent on LMAN2L transport, thereby leading to viral evasion of host immune surveillance (44). Overall, the LMAN2L gene is involved in protein quality control and transport in the early secretory pathway of cells, playing a crucial role in nervous system development, airway infection defense, tissue damage repair, viral infection, and immune regulation. Our research results indicate that LMAN2L is a protective factor for CRC. Based on the biological function of the LMAN2L gene, we reasonably speculate that dysfunction of the LMAN2L gene may lead to the accumulation of misfolded proteins, thereby triggering ER stress response, or by inhibiting the body’s anti-tumor immunity, providing a favorable microenvironment for tumor cell growth. The specific association and mechanism of action of this gene with CRC still require validation through extensive experimental research.

This study identified CTSF, PCSK7, LYZ, and LMAN2L as critical regulatory genes in colorectal cancer, demonstrating their multifaceted clinical potential in diagnosis, therapeutic intervention, and prognostic evaluation. The bidirectional expression pattern of CTSF (downregulated in renal/gastric cancers vs. upregulated in cervical cancer/NSCLC brain metastases) positions it as a disease-specific biomarker: serum CTSF levels combined with imaging could enhance diagnostic accuracy for NSCLC brain metastases, while tissue CTSF expression may predict recurrence risk in clear-cell renal cell carcinoma patients. PCSK7’s dual roles in metabolic regulation and immune modulation suggest precision therapeutic strategies—hepatocyte-targeted ASO agents suppressing its pro-lipogenic function have alleviated NAFLD phenotypes in murine models, potentially extendable to metabolism-associated CRC subtypes, while PCSK7 inhibitors synergizing with anti-PD-1 therapy reversed T-cell exhaustion in colon cancer organoids. LYZ-mediated gut microbiota regulation highlights preventive potential, where dietary interventions (e.g., lysozyme-fortified probiotics) may disrupt the “*Fusobacterium nucleatum*-lysozyme-tumor microenvironment” axis, and preoperative recombinant lysozyme supplementation may mitigate chemotherapy-induced intestinal damage in elderly patients. LMAN2L’s pivotal role in ER quality control enables prediction of chemotherapy sensitivity through ER stress biomarkers, while AAV vector-based gene supplementation shows promise in rectifying proteostatic imbalance, as preliminarily validated in COPD models. Future multi-center cohorts should establish clinical thresholds for these biomarkers, coupled with developing spatiotemporal-specific regulatory strategies to optimize therapeutic safety profiles.

### Limitations

Our study inevitably possesses certain limitations. The GWAS and eQTL/pQTL data were derived from diverse European populations (Finnish and Icelandic cohorts), which might restrict the cross-population generalizability due to differences in population genetic structures. Although we employed stringent linkage disequilibrium correction (r^2^ < 0.1, clumping distance > 10,000 kb) to minimize heterogeneity interference, the strength of genotype–phenotype associations might still exhibit population-specific biases across different cohorts. Additionally, the mtRNA filtering threshold (> 10%) used in data preprocessing might lead to the loss of mitochondrial high-activity immune cell subgroups. Moreover, the existing eQTL data primarily based on whole blood or intestinal tissue groups, while the pQTL data were derived from the plasma proteome, present disparities with the spatiotemporal specificity of local protein expression in the colorectal cancer cell microenvironment. This cross-tissue type data integration could introduce biological-level misjudgments. Although the HEIDI test was used to exclude obvious pleiotropic loci, genetic instrumental variables might still affect disease outcomes through non-target pathways, such as regulating adjacent genes or epigenetic modifications. To comprehensively assess potential pleiotropy, we performed additional sensitivity analyses. The MR-Egger intercept test showed no evidence of directional pleiotropy (all p > 0.05), and the MR-PRESSO global test indicated no significant outliers or heterogeneity (p > 0.05). Furthermore, Cochran’s Q test demonstrated consistent results across instrumental variables (p > 0.05), suggesting minimal horizontal pleiotropic effects. Although these analyses help address major biases, it is important to acknowledge that hidden effects through other biological pathways might still affect the causal conclusions.

Despite these limitations, genetic heterogeneity across populations offers a natural framework to explore regulatory mechanisms in diverse ethnic groups. For instance, if genes like CTSF and PCSK7 maintain stable associations in populations with significantly different genetic backgrounds, this substantially strengthens their evidence as conservative therapeutic targets. Moreover, the potential pleiotropy of genetic instrumental variables, even after rigorous screening (F > 10), might point to “hub genes” with multi-tissue regulatory functions (such as LMAN2L’s broad role in the protein secretion pathway), which often play crucial roles in complex disease networks. These seemingly limiting characteristics essentially anchor more translationally potent directions for subsequent research.

## Conclusion

By combining single-cell analysis, eQTL analysis, pQTL analysis, and SMR analysis, we have identified four potential disease targets (CTSF, PCSK7, LYZ, LMAN2L), providing new potential targets and ideas for the development of gene and protein regulation-related drugs for CRC, which is of great significance for improving the prevention, diagnosis, and treatment of colorectal cancer.

## Supplementary Information

Supplementary file 1

Supplementary file 2

## Data Availability

All data used in the text are included in the text or can be found in the supplementary material. Also available from the corresponding author at zhangt2010@gxtcmu.edu.cn.
