# Supplementary figures and images for: Integrating single-cell with transcriptome-proteome Mendelian randomization reveals colorectal cancer targets
Source: Discov Oncol. 2025 May 17;16:794. doi: 10.1007/s12672-025-02636-7 (PMC12085524; doi:10.1007/s12672-025-02636-7)

Normal

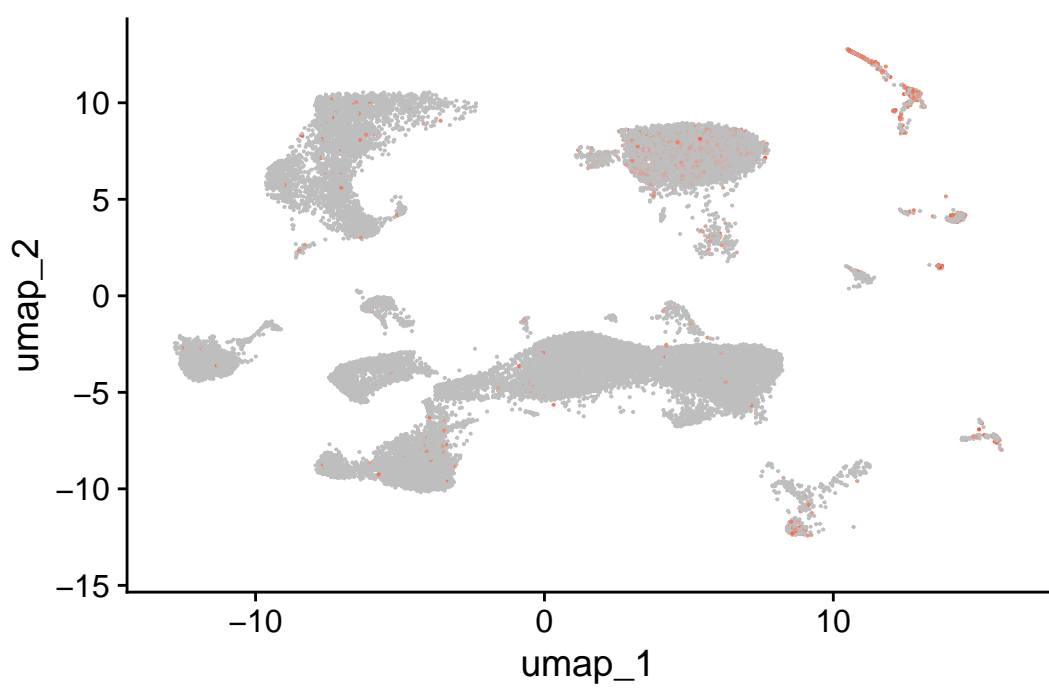

Tumor

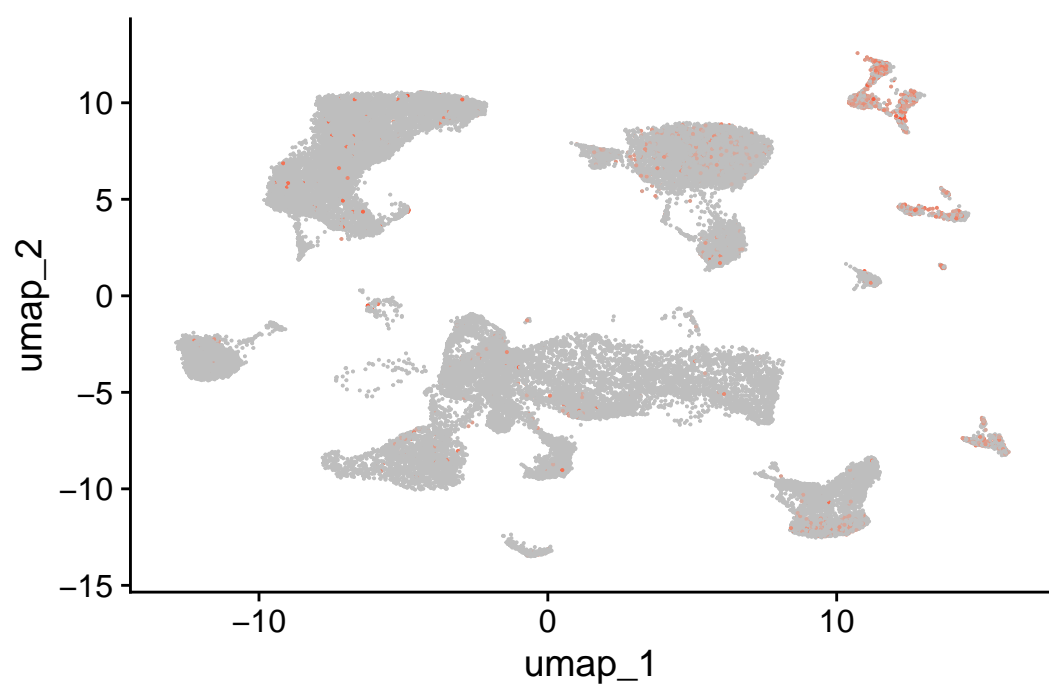

CTSF

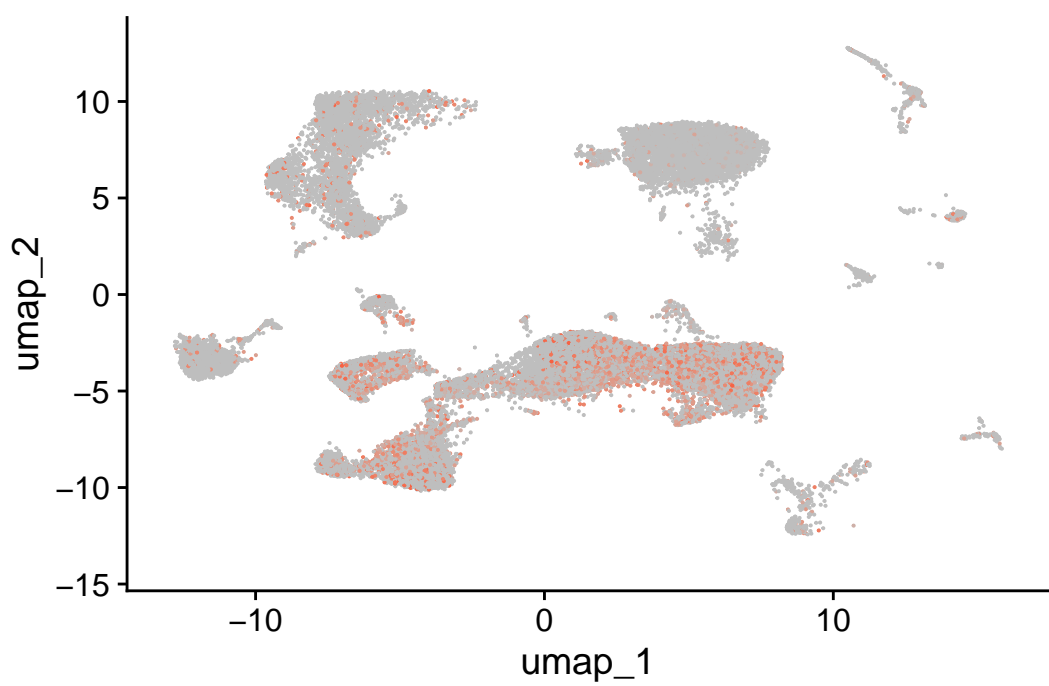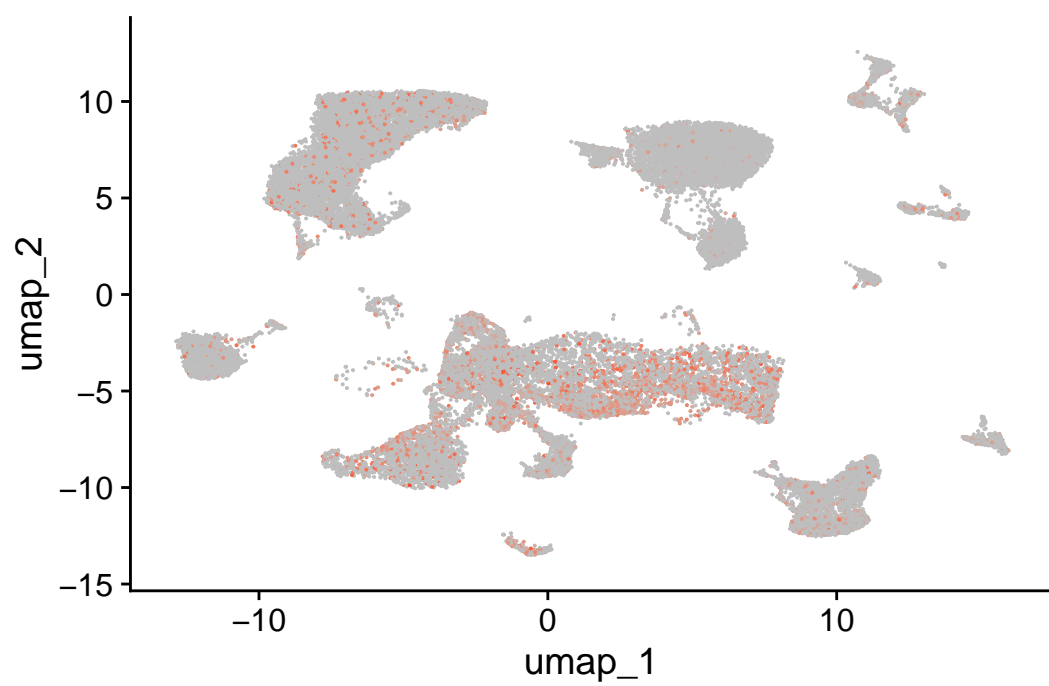

PCSK7

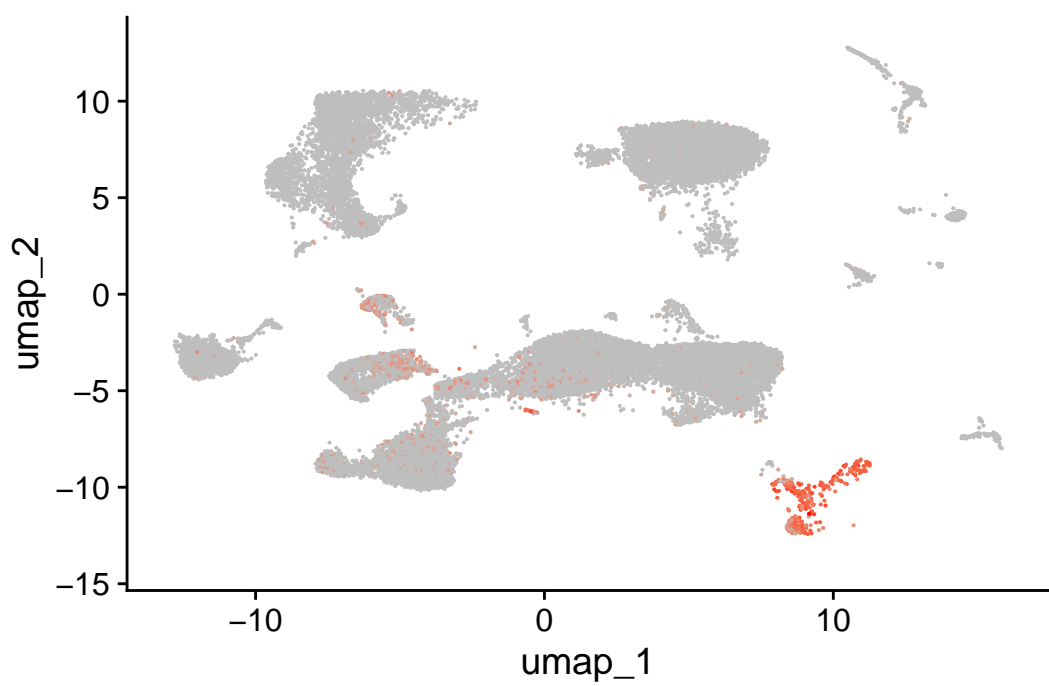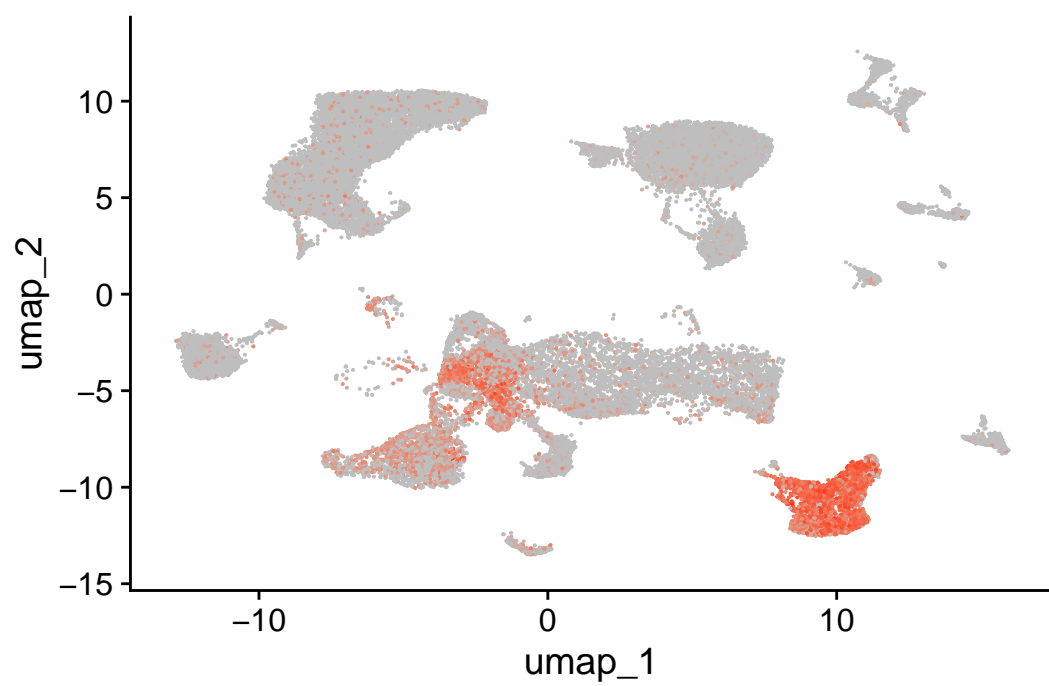

LYZ

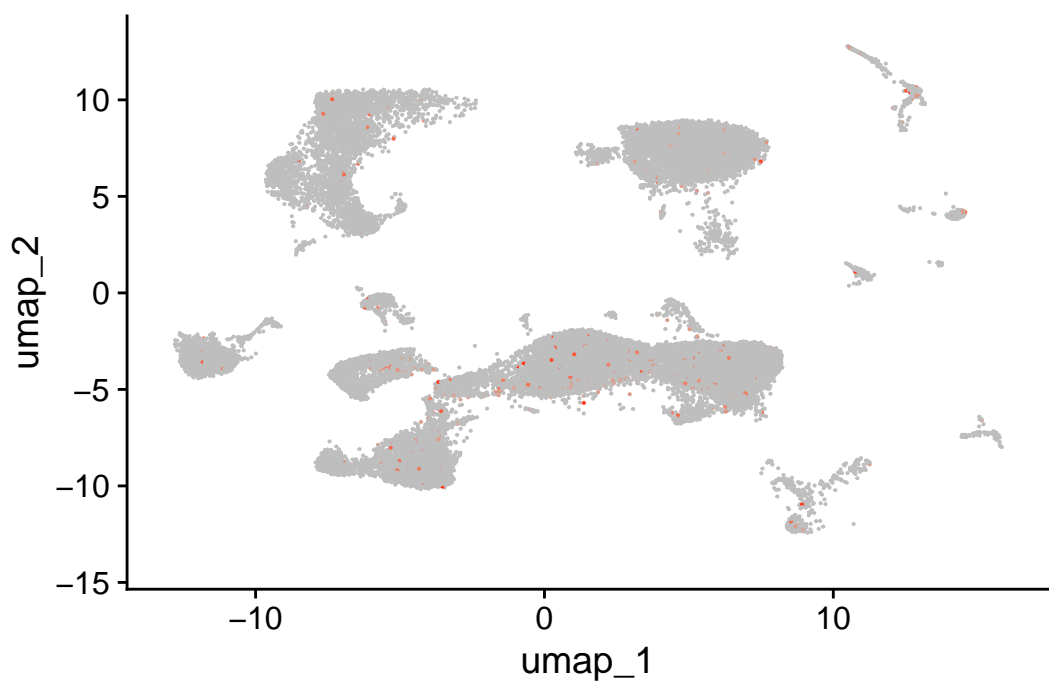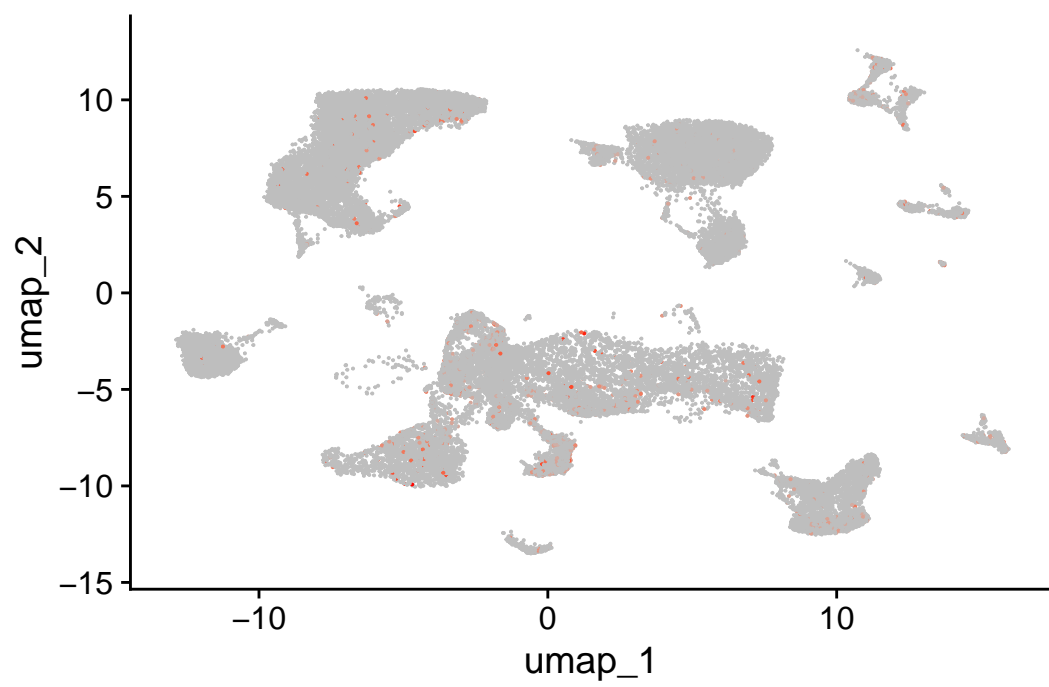

LMAN2L

Supplement: Supplementary file 1 [file 12672_2025_2636_MOESM1_ESM.pdf]
